# Opportunities and obstacles in non-invasive brain stimulation

**DOI:** 10.3389/fnhum.2024.1385427

**Published:** 2024-03-18

**Authors:** Jake Toth, Danielle Lauren Kurtin, Méadhbh Brosnan, Mahnaz Arvaneh

**Affiliations:** ^1^Automatic Control and Systems Engineering, Neuroscience Institute, Insigneo Institute, University of Sheffield, Sheffield, United Kingdom; ^2^Department of Brain Sciences, Imperial College London, London, United Kingdom; ^3^School of Psychology, University College Dublin, Dublin, Ireland; ^4^Department of Experimental Psychology, University of Oxford, Oxford, United Kingdom; ^5^Oxford Centre for Human Brain Activity, Wellcome Centre for Integrative Neuroimaging, Department of Psychiatry, University of Oxford, Oxford, United Kingdom; ^6^Turner Institute for Brain and Mental Health and School of Psychological Sciences, Monash University, Melbourne, VIC, Australia

**Keywords:** brain stimulation, tES, TMS, tPBM, TUS, tDCS, FUS, neurotechnology

## Abstract

Non-invasive brain stimulation (NIBS) is a complex and multifaceted approach to modulating brain activity and holds the potential for broad accessibility. This work discusses the mechanisms of the four distinct approaches to modulating brain activity non-invasively: electrical currents, magnetic fields, light, and ultrasound. We examine the dual stochastic and deterministic nature of brain activity and its implications for NIBS, highlighting the challenges posed by inter-individual variability, nebulous dose-response relationships, potential biases and neuroanatomical heterogeneity. Looking forward, we propose five areas of opportunity for future research: closed-loop stimulation, consistent stimulation of the intended target region, reducing bias, multimodal approaches, and strategies to address low sample sizes.

## 1 Introduction

The human brain, comprising approximately 80 billion neurons and a similar number of glial cells (Herculano-Houzel, 2009), is a highly complex organ. The brain consumes 20% of the body's metabolic budget while constituting only 2% of its mass (Sokoloff, 1960). The outsized cardiovascular intake of the brain sustains its many parallel processes, estimated at (0.18–6.4) × 10^14^ traversed edges per second (Katja, 2015). Neuromodulatory interventions aim to deliver targeted perturbations of these neural processes, identifying the optimal targets and methods poses a significant challenge. Additionally, neuromodulation must take into account the cerebrospinal fluid, sulci and gyri, meninges, skull, and scalp in which the brain is ensconced. Yet the ability to modulate brain activity without surgery, both in the cortex and in deep brain structures, has been demonstrated through a broad spectrum of approaches (Polańıa et al., 2018).

Non-invasive brain stimulation (NIBS) is a promising approach for improving brain health and quality of life. Moreover, when appropriately configured it presents minimal risks and costs when compared to invasive brain stimulation (Najib and Horvath, 2014; Bikson et al., 2016; Cassano et al., 2022; Qin et al., 2023). Greater patient acceptability facilitates the translation of NIBS to a broader range of applications. For instance, patients with moderate Parkinson's disease consider invasive deep-brain stimulation to be a last resort (Sperens et al., 2017). In a survey of the general public, ultrasound and magnetic stimulation were preferred over pharmaceuticals and implants as an intervention for mental health (Atkinson-Clement et al., 2024).

Applications of NIBS include personalized, intermittent theta-burst transcranial magnetic stimulation (TMS) which has been approved by the Federal Drug Administration (Tarr, 2023) and boasts a 79% remission rate for patients with treatment-resistant depression (Cole et al., 2022). Transcranial electrical stimulation (tES) is being trialed by the National Health Service in the United Kingdom for the treatment of depression (Medtech innovation briefing, 2023). The treatment of drug addiction has promise in preliminary trials using low-intensity focused ultrasound stimulation (LIFUS) (Mahoney et al., 2023). NIBS has also been used for the reduction of chronic pain (Che et al., 2021) and the enhancement of attention (Brosnan et al., 2018) and memory (Grover et al., 2022) in the aging brain.

NIBS shows promise as a therapeutic intervention for various neurological and psychiatric conditions for both its wide-ranging neuromodulatory capabilities and the accessibility of large-scale manufacturing. The manufacturing of NIBS devices relies largely on the existing electronics manufacturing industry, as a result, it has the potential to scale quickly. This could potentially facilitate adoption curves akin to those seen for blood oxygen sensors or consumer electronics products within the respective patient groups. At this scale and speed, NIBS could have a substantial, translational impact. However, the realization of NIBS's potential is contingent upon surmounting several significant barriers. In this work, we introduce different approaches to NIBS and their mechanisms of action, describe major challenges facing the field, and explore opportunities to overcome them.

## 2 Approaches to non-invasive brain stimulation

**Figure 1:**
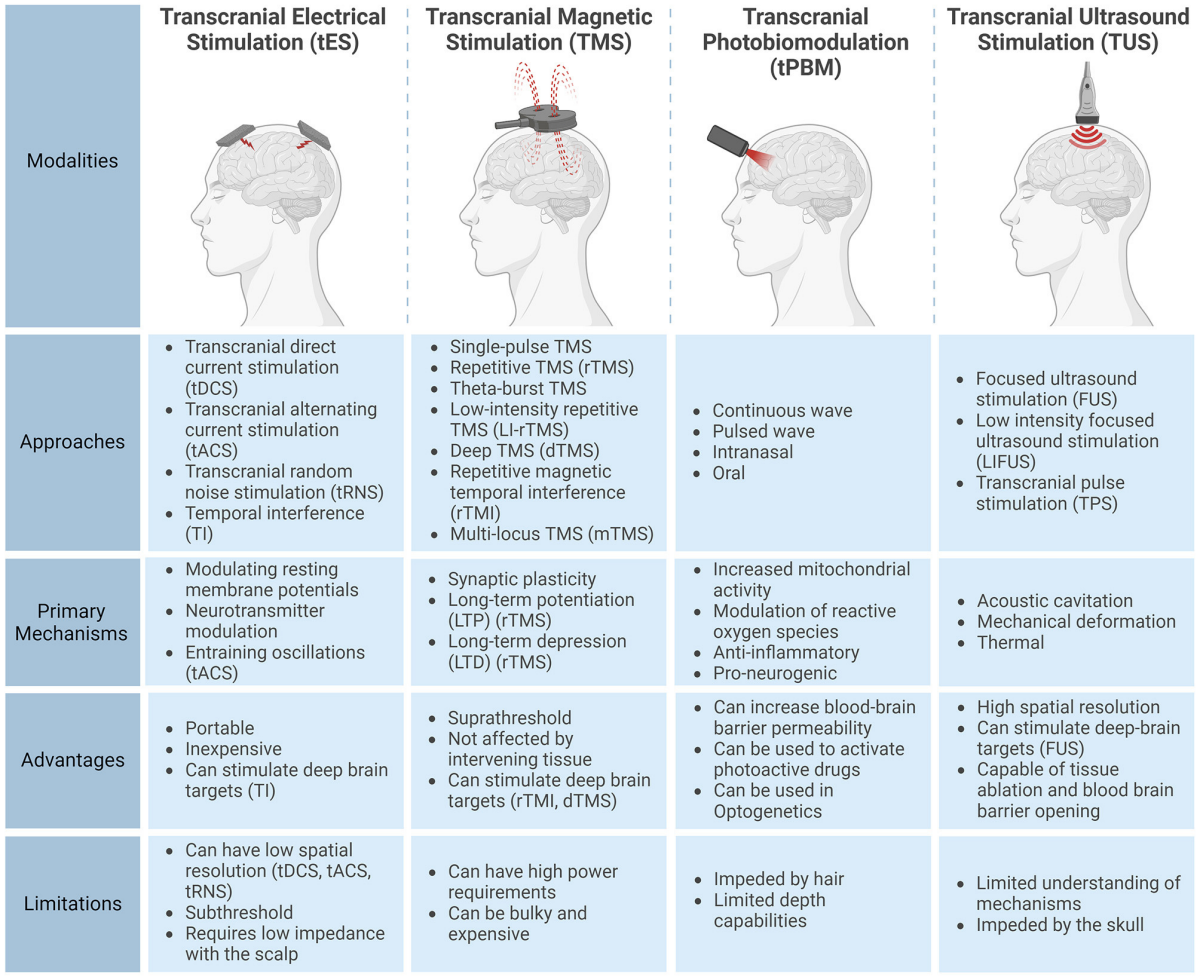
Characteristics of the four non-invasive brain stimulation modalities: transcranial electrical stimulation (tES), transcranial magnetic stimulation (TMS), transcranial photobiomodulation (tPBM), and transcranial ultrasound stimulation (TUS). This figure outlines common subtypes, primary mechanisms, advantages, and limitations for each modality. Created with BioRender.com.

The four primary approaches to non-invasive brain stimulation are transcranial electrical stimulation (tES), transcranial magnetic stimulation (TMS), transcranial photobiomodulation (tPBM) and transcranial ultrasound stimulation (TUS). Figure 1 provides an overview of their respective characteristics.

Transcranial electrical stimulation (tES) passes a current (typically 1-4mA) through active and return electrodes (Thair et al., 2017). Stimulation is typically delivered via two electrodes, although more electrodes can enhance stimulation focality (Datta et al., 2009). Transcranial direct current stimulation (tDCS), a type of tES, modulates neuronal excitability without reaching the threshold necessary to trigger neuronal depolarizations (Bikson et al., 2004). This alteration of membrane potentials has been shown to occur through several mechanisms, including decreased γ-aminobutyric acid (GABA) concentrations (Stagg et al., 2011) and increased glutamate and glutamine concentrations (Hunter et al., 2015) with effects modulated by sodium channels (Nitsche et al., 2003; Alagapan et al., 2016; Reed and Cohen Kadosh, 2018).

tES also comes in other forms, such as Transcranial Alternating Current Stimulation (tACS) which utilizes sinusoidal currents thought to entrain brain oscillations (Antal et al., 2008; Kanai et al., 2008). Additionally, Transcranial Random Noise Stimulation (tRNS) is the random sampling of currents from a bell-shaped curve (Terney et al., 2008). The mechanisms for tRNS are unclear, but they may involve stochastic resonance (Stacey and Durand, 2000). In contrast to tDCS, aftereffects of tRNS are not NDMA receptor-dependent and can be suppressed by benzodiazepines, suggesting an alternative mechanism (Chaieb et al., 2015).

Temporal Interference (TI) stimulation employs the convergence of two or more high-frequency (kHz) currents to create a low-frequency envelope that selectively modulates brain activity. Importantly, the high-frequency carrier currents, though present, do not impact neuronal activity due to the low-pass filtering effect of neuronal membranes (Hutcheon and Yarom, 2000). This generates a focused electric field (E-field) that oscillates at the difference in frequency of these high-frequency waves (Grossman et al., 2017). Notably, recent human studies have successfully demonstrated TI in humans, including the targeted modulation of the hippocampus (Violante et al., 2023).

Transcranial magnetic stimulation (TMS) uses electromagnets to induce currents within the brain, producing supra-threshold E-fields on the order of hundreds of V/m (Bijsterbosch et al., 2012) compared to less than 1V/m with tES (Evans et al., 2020). Approaches to TMS includes single-pulse TMS for the exploration of brain function and repetitive TMS (rTMS) used to induce effects lasting longer than the stimulation period (Klomjai et al., 2015). TMS can induce long-term potentiation (LTP) through the unblocking of post-synaptic NDMA receptors blocked by magnesium ions resulting in an influx of calcium ions in post-synaptic neurons (Cooke and Bliss, 2006). Additionally, long-term depression (LTD) can be induced through the slow flow of calcium ions from lower-frequency stimulation (Chervyakov et al., 2015). TMS can also alter gene expression and enzyme production along with a multitude of other effects (Chervyakov et al., 2015). Notably, rTMS effects are frequency-dependent, with inhibition below 1Hz and facilitation above 5Hz, although this generalization is disputed (Prei et al., 2023).

The application of TMS involves diverse pulse patterns, tailored to specific purposes. One notable example is theta-burst TMS, showing promise in treating major depressive disorder. This method employs short bursts of 50Hz stimulation to mimic the effects of LTP and LTD, thought to induce synaptic plasticity while offering shorter, lower-intensity procedures than traditional rTMS (Chung et al., 2015). Low-Intensity Repetitive TMS (LI-rTMS) uses an order of magnitude weaker magnetic field strengths (1-150mT) and remains subthreshold, unlike typical TMS that can evoke motor potentials (Barker et al., 1985; Moretti and Rodger, 2022). Furthermore, deep brain regions can be stimulated with deep TMS (dTMS) using a specialized coil known as the Hesed coil (Zangen et al., 2005). Additionally, repetitive magnetic temporal interference (rTMI), which utilizes the electrotemporal interference of high-frequency TMS holds promise for selectively modulating deep-brain targets (Khalifa et al., 2023). Finally, multi-locus TMS (mTMS) is an approach that uses an array of electronically controlled coils, allowing for the shifting and re-orienting of the induced E-field (Koponen et al., 2018).

Transcranial Photobiomodulation (tPBM) commonly employs near-infrared light (600 nm–1,100 nm) to modulate brain activity (Salehpour et al., 2018). Light that reaches the brain is absorbed by chromophores, particularly Cytochrome c oxidase, increasing mitochondrial activity and Adenosine triphosphate production. This also modulates reactive oxygen species and the release of calcium ions, activating transcription factors for long-term cellular effects (Caldieraro and Cassano, 2019). Resultant biological responses include improved oxidative metabolism and increased blood flow (Caldieraro and Cassano, 2019). tPBM is also thought to have anti-inflammatory and pro-neurogenic effects (Cassano et al., 2016). However, the absorption of light by hair limits tPBM applications outside of the forehead region, intranasally or through the oral cavity (Salehpour et al., 2018; Askalsky and Iosifescu, 2019). While tPBM traditionally uses a continuous wave, recent work explores pulsed wave tPBM, allowing for short, high-power pulses (Tang et al., 2023).

Thus far conclusive evidence regarding the efficacy of tPBM for depression and dementia is yet to emerge (Salehpour et al., 2021; Vieira et al., 2023). However, there are encouraging results regarding the effects of tPBM on cognitive abilities and brain function during aging (Dole et al., 2023). Aside from modulating neural activity directly, a picosecond 532nm pulse has been observed as temporarily improving blood-brain barrier permeability (Li et al., 2021), enhancing the delivery and efficacy of paclitaxel in mice with glioblastoma (Cai et al., 2023). Additionally, advances in photopharmacology offer the potential to precisely control pharmaceuticals within the brain using light (Velema et al., 2014).

Transcranial ultrasound stimulation (TUS) uses high-frequency sound waves for non-invasive neural modulation. It is employed in various applications, including the increase of blood-brain barrier permeability via cavitation (Hynynen et al., 2001) and tissue ablation through heating (Martin et al., 2009). Alternatively, low-intensity ultrasound has been demonstrated to modulate brain activity. Examples include the attenuation of thermal pain sensitivity (Badran et al., 2020) and sensory-evoked brain oscillations (Legon et al., 2014). Focused ultrasound stimulation (FUS) uses out-of-phase high-frequency waves to stimulate deep brain regions with millimeter precision (Ghanouni et al., 2015). Low-intensity focused ultrasound (LIFUS) is the application of ultrasound stimulation at low intensities to modulate neuronal activity without causing tissue damage. Thermal effects are thought to be negligible from LIFUS as tissue temperatures rise by less than 0.1°C. Non-thermal effects from LIFUS include stable acoustic cavitation, which involves pressure-induced oscillations of gas-filled microbubbles and influencing neuron excitability via membrane deformation. Additionally, LIFUS can alter ion channel permeability and membrane characteristics through mechanical energy, impacting ion flux and neuronal membrane discharge from mechanosensitive ion channels. These complex interactions are thought to contribute to the neuromodulatory effects of LIFUS (Fomenko et al., 2018). Transcranial pulsed stimulation (TPS) employs short pulses of ultrasound lasting 3μs. These short pulses mitigate brain heating and preliminary evidence suggests cognitive benefits in Alzheimer's patients (Beisteiner et al., 2020).

## 3 Obstacles to effective non-invasive brain stimulation

**Figure 2:**
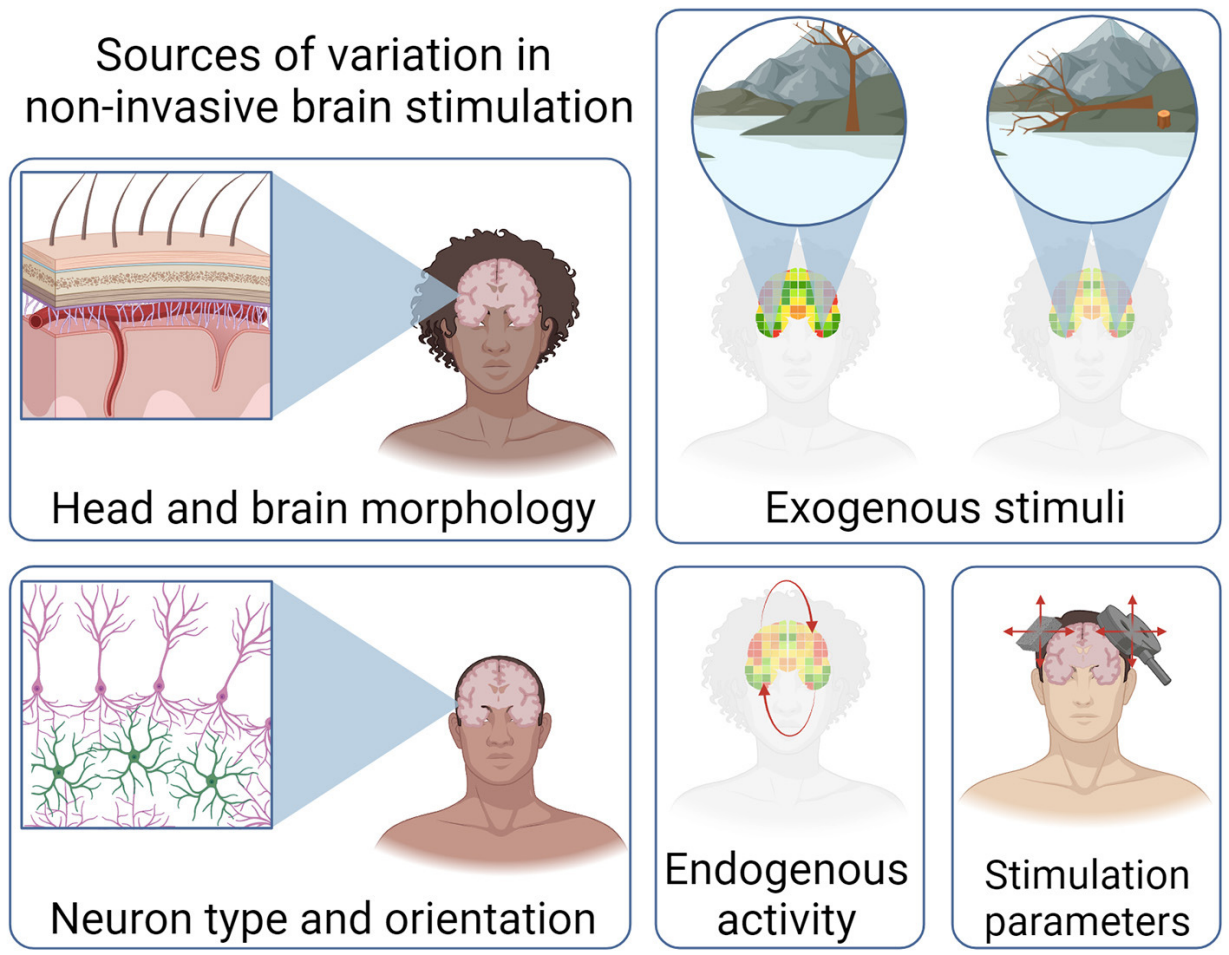
Illustration of the sources of variation that influence the efficacy of non-invasive brain stimulation. Variations in head and brain morphology, exogenous stimuli, neuron type and orientation, as well activity and stimulation parameters. Created with BioRender.com.

The brain can be considered a dynamical, emergent complex system (Turkheimer et al., 2022). Put simply, ongoing brain function is a result of the interplay between exogenous stimuli/perturbations and endogenous processes and goals. The brain presents both stochastic and deterministic characteristics, underpinning the difficulty in developing a mechanistic understanding of brain function and behavior. While the causal perturbations of NIBS facilitate the characterization of neural mechanisms, the complex, individual nature of brain structure and function makes the optimization of NIBS challenging. These sources of variation are illustrated in Figure 2.

One source of complexity is the inter-individual variation in head and brain morphology. For instance, skull thickness (Lillie et al., 2016) and cortical thickness, volume, and surface area are negatively correlated with age (Lemaitre et al., 2012). In adults, ventricles within the brain become larger with age and both white and gray matter volumes are reduced (Bethlehem et al., 2022). These inter-individual and intra-individual variations are particularly consequential in tES. For example, current flow modeling has demonstrated that identical tES parameters result in an over 100% difference in the intensity of the E-field generated in the motor cortex (Evans et al., 2020). There is also the potential for unintentional stimulation of off-target regions, especially when individualized modeling is not performed (Soleimani et al., 2023).

The correlation between the applied stimulation dose and the resulting response remains unclear. In tPBM, a biphasic dose-response curve is suggested (Yang M. et al., 2021). Conversely, a positive relationship between the dose of TUS applied to the lateral geniculate nucleus and the subsequent suppression of visually evoked potentials has been observed (Mohammadjavadi et al., 2022). Regarding tDCS, a simple linear dose-response relationship may be an oversimplification (Esmaeilpour et al., 2018). Dose-response relationships across NIBS remain unclear, likely due to a complex interplay of factors including the functional state of the brain during stimulation.

The functional state of the brain during stimulation is an important area of consideration when optimizing NIBS (Bradley et al., 2022). For instance, responses to tES are associated with preexisting concentrations of the neurotransmitters GABA and glutamate (Filmer et al., 2019). However, the specific nature of the brain-state dependent dose-response relationship in tES remains inadequately understood (Lee et al., 2021). Similarly, FUS differentially affects active and resting neurons (Yang P.-F. et al., 2021). The use of electroencephalography (EEG) for measuring TMS evoked potentials has proven effective in optimizing stimulation orientation in the left pre-supplementary motor area (Tervo et al., 2022). Additionally, it enables real-time adjustment of stimulation protocols to control the efficacy of plasticity induction in the motor cortex (Zrenner et al., 2018). As with NIBS more broadly, applying these state-dependent techniques beyond the motor system is an additional challenge.

The type and orientation of neurons being stimulated is another complicating factor in elucidating dose-response relationships. For instance, when FUS is applied, neurons exhibit varying responses to different pulse frequencies, influenced by their distinct action potential waveforms and genotypes (Yu et al., 2021). An important factor of the efficacy of tES is the orientation of neurons relative to the E-field generated. Additionally, neurons with highly-branched axons are less sensitive to the relative direction of the E-field (Chung et al., 2022). Interestingly, stimulation itself can also change structural components of the brain. For instance, subthreshold rTMS alters the dendritic spine structure in pyramidal neurons in mice (Tang et al., 2021) and static magnetic stimulation through the use of a permanent magnet has been shown to decrease the length of the axon initial segment in cell cultures (Beros et al., 2022). In the development of stimulation configurations, modeling current flow allows for a comprehensive understanding of the interplay between stimulation montage, E-field, and neural orientation (Lee et al., 2021). Further developments in multi-scale modeling may elucidate these relationships.

It is common practice in NIBS to focus on stimulating a single brain region, even though many targeted behaviors involve a distributed network of activity. For instance, in depressed populations, the prefrontal cortex is often targeted, but depression symptoms are linked to activity changes in various brain regions including the thalamus and hippocampus among others (Zhang et al., 2018). Consequently, achieving optimal stimulation parameters may necessitate simultaneous stimulation of multiple targets. Although progress has been made in this direction (Fischer et al., 2017; Corlier et al., 2021), it remains a relatively unexplored area.

There are a multitude of other factors that may impact the efficacy of NIBS, from circadian rhythms (Vergallito et al., 2022) to caffeine (Zulkifly et al., 2021) and broader genetic predispositions (Hasan et al., 2013). Brain temperature variations linked to age, sex, and menstrual cycle (Rzechorzek et al., 2022) may also impact NIBS. As with all experimental studies, excluding common disorders like dyslexia or ADHD limits the generalizability of findings from healthy controls, given that 15-20% of the population is neurodivergent (Doyle, 2020). Further, underdiagnosis in certain groups, such as females with ADHD (Young et al., 2020), may result in the inadvertent inclusion of undiagnosed neurodivergent individuals. Overly broad exclusion criteria can introduce population bias; insufficient accommodations for individuals who wear head coverings or exhibit sensory processing sensitivity may hinder diverse participation. Many studies, excluding those focusing on disorders prevalent in older adults, predominantly involve young participants and are conducted in a limited number of countries (Sun et al., 2022; Medeiros et al., 2023; Wang et al., 2023).

## 4 Areas of opportunity and future research directions

The effects of NIBS are state-dependent, making adaptive stimulation attuned to brain dynamics crucial for maximizing the potential of NIBS (Bergmann, 2018). In a closed-loop NIBS system, brain activity is continuously monitored and compared to a desired state, and stimulation parameters are adjusted in real-time to optimize the therapeutic effects. To facilitate a greater understanding of the mechanisms and predictors of NIBS efficacy, multi-scale models will be informed by multi-scale imaging from synapse to lobe (D'Angelo and Jirsa, 2022). Ultimately, state-dependent neuromodulation could be delivered in more wearable form. EEG captures ongoing brain states non-invasively and has enabled tACS delivered to match the phase and frequency of slow-wave oscillations to enhance long-term generalized memory consolidation post-sleep (Ketz et al., 2018). Additionally, high-definition diffuse optical tomography provides high spatial resolution capture of hemodynamics in the cortex (Vidal-Rosas et al., 2021) and Optically Pumped Magnetometers-Magnetoencephalography (OPM-MEG) enables high spatial and temporal resolutions and sensitivity (Brookes et al., 2022). Optimizing stimulation parameters based on real-time brain state is both necessary and attainable.

Another central limitation is ensuring consistent stimulation of the intended target. Electrode montages are chosen to stimulate a target region, in practice the position and intensity of stimulation in the brain varies between individuals and sessions. Precise sensor and stimulator positioning is crucial for accuracy (Shirazi and Huang, 2019; Caulfield et al., 2022). Achieving such precision involves structural imaging, simulation, and neuronavigation, a resource-intensive process. More scalable approaches are needed, potentially combining subject-specific MRI approximated with generative modeling (Tudosiu et al., 2022) and utilizing smartphone-based 3D head scanning (Everitt et al., 2023). Additionally, techniques such as electrical impedance tomography and pulse-echo ultrasound can estimate the electrical and acoustic properties of the head, respectively (Fernández-Corazza et al., 2017; He et al., 2021). Notably, advances in low-field MRI may offer more accessible structural imaging with 64mT mobile scans approaching the quality of 3T scans (Lucas et al., 2023). Statistical techniques that account for inter-individual variability, such as mixed-model analyses or cluster analysis, may also be used as opposed to the standard population-based approaches (Vergallito et al., 2022).

One source of bias in NIBS research is rooted in the challenges associated with gaining access to the scalp, especially when dealing with long, thick, and/or curly hair (Louis et al., 2022). There is a recognized bias in neuroscience concerning race and sex (Kwasa et al., 2023). Addressing these biases could involve implementing inclusive training and recruitment procedures. Technological solutions, such as 3D-printed electrodes designed for various hair types, may also contribute to mitigating these challenges (Xing and Casson, 2023). Additionally, offering additional remuneration for participants with hair that requires more time for preparation and washing is a consideration. To foster more representative research samples, further open-sourcing of datasets containing demographic information is recommended for the benefit of future researchers.

The efficacy of NIBS could potentially be enhanced by combining stimulation modalities as each approach offers unique benefits and mechanisms of action. For instance, priming with tDCS before applying TMS may produce more robust effects (Hurley and Machado, 2017). Similarly, concerning closed-loop NIBS, multi-modal approaches for reading brain activity can be more effective at classifying brain states as has been demonstrated by the combination of EEG and functional Near Infrared Spectroscopy to improve seizure detection accuracy (Sirpal et al., 2019). Multi-modality may potentially extend to non-invasive optogenetics, with recent work showing the delivery of viral vectors facilitated by FUS to increase blood-brain barrier permeability, followed by channel activation using light in mice (Pouliopoulos et al., 2022). As such, it may be pertinent for further advancement of multi-modal sensing and stimulation.

Finally, statistical power emerges as a noteworthy concern in NIBS research. The average sample size in studies involving tDCS and TMS is 22, leading to a situation where NIBS studies may miss 50% of true positive results (Mitra et al., 2019). While practical constraints often limit the sample size of initial studies, enhancing statistical power through adequately powered studies is crucial. In instances where inter-individual variability is not accounted for through brain imaging and simulation, broader utilization of “N-of-1” trials with a single individual with openly available data, might be a pertinent approach for NIBS research as it is with personalized medicine more broadly (Schork, 2015). The aggregation of such studies has the potential to offer valuable insights beyond the conventional population-based approach as the inter-individual source of variability is not a concern. This could also allow for greater use of longitudinal study designs, that investigate the long-term effects of multi-session stimulation.

## 5 Discussion

In this paper, we have explored the multifaceted landscape of NIBS, delving into the four main modalities: tES, TMS, tPBM, and TUS. Each modality possesses unique attributes, mechanisms and challenges, contributing differently to the field of neurostimulation. Yet all must deal with the inherent complexities and challenges associated with individual morphological and physiological variations, as well as the dynamic nature of the brain and the subsequent state-dependent nature of stimulation.

Looking forward, this paper highlights several avenues for advancing NIBS. The development of closed-loop systems that integrate real-time brain state monitoring with stimulation holds the promise of enhancing efficacy. Overcoming the challenge of consistently stimulating the intended brain regions across individuals necessitates innovative approaches, potentially leveraging advances in imaging technologies and computational modeling. Addressing biases in NIBS research and its applications, including those related to race, sex, and neurodiversity, is critical for ensuring the broad applicability and effectiveness of these technologies. Furthermore, exploring the synergistic potential of multi-modal NIBS approaches may improve outcomes. Finally, tackling the issue of underpowered studies through adequately sized trials, personalization and appropriate statistical methods. Implementing these recommendations could help propel NIBS toward achieving its full therapeutic potential across diverse populations.

## Data availability statement

The original contributions presented in the study are included in the article/supplementary material, further inquiries can be directed to the corresponding author.

## Author contributions

JT: Conceptualization, Visualization, Writing – original draft, Writing – review & editing. DK: Writing – review & editing. MB: Supervision, Writing – review & editing. MA: Supervision, Writing – review & editing.
